# Characterization of the Complete Mitochondrial Genome and Evaluation of COI Barcoding in *Philonis inermis* (Coleoptera: Curculionidae: Cryptorhynchinae) Using Genome Skimming

**DOI:** 10.12688/f1000research.170584.3

**Published:** 2026-04-24

**Authors:** Alejandra Clavijo-Giraldo, Sandra Uribe Soto, Andrés Gómez-Palacio

**Affiliations:** 1 Grupo de Investigación en Sistemática Molecular - GSM, Universidad Nacional de Colombia, Medellín, Colombia; 2 Laboratorio de Investigación en Genética Evolutiva - LIGE, Universidad Pedagogica y Tecnologica de Colombia, Tunja, Boyaca, Colombia; 3 Grupo de Estudios en Genética y Biología Molecular - GEBIMOL, Universidad Pedagógica y Tecnológica de Colombia, Tunja, Boyacá, Colombia

**Keywords:** Neotropical weevils; mitochondrial genome; COI barcode; Curculionidae; Passiflora foetida; biological control

## Abstract

**Background:**

*Philonis inermis* is a Neotropical stem-galling weevil specialized on the invasive vine *Passiflora foetida* and represents a promising candidate for biological control. However, no genomic or barcoding data have previously been available for this genus, limiting its taxonomic resolution and risk assessment potential.

**Methods:**

We used shallow whole-genome sequencing of two individuals reared under controlled conditions to assemble, annotate, and compare the complete mitochondrial genome of *P. inermis* with other Cryptorhynchinae. BUSCO analysis was performed to recover nuclear single-copy orthologs and additional multicopy markers. *Cytochrome c* oxidase subunit I (COI) sequences from 20 Colombian specimens were analyzed together with 24 Cryptorhynchinae barcodes from GenBank to evaluate intra- and interspecific divergence.

**Results:**

The *P. inermis* mitogenome is 15,120 bp in length, AT-rich (77.0%), and contains 36 genes, including 13 protein-coding genes, 21 tRNAs, and two rRNAs. The tRNA-Ile was not detected, likely obscured within the variable control region, as reported for other cryptorhynchine weevils. Phylogenetic analysis based on mitogenomic sequences placed *P. inermis* as a well-supported clade closely related to *Eucryptorrhynchus.* COI barcode analysis revealed extremely low intraspecific divergence (pairwise K2P ≤ 0.006) and a pronounced barcode gap distinguishing *P. inermis* from other Cryptorhynchinae species. Genome-skimming assemblies yielded 196 single-copy orthologs, 28 duplicated BUSCOs, and a rich set of multicopy nuclear markers, including extensive rRNA fragments (18S, 28S, 5.8S, 16S) and core histones (H2A, H2B, H3, H4), which are provided as extended data for future phylogenomic applications.

**Conclusion:**

This study presents the first complete mitochondrial genome for the genus *Philonis* and demonstrates the utility of COI barcoding for the current molecular identification of *P. inermis*, in a context where comparative mitogenomic data remain scarce. These genomic resources provide a foundation for future integrative taxonomic, comparative, and evolutionary studies, and support further evaluation of *P. inermis* as a potential biological control agent against *P. foetida.*

## 1. Introduction

Within Cryptorhynchinae subfamily (Coleoptera: Curculionidae), the Neotropical genus *Philonis* represents an underexplored lineage of stem-galling weevils that are tightly associated with species of *Passiflora* (Passifloraceae). Among them, *Philonis inermis* has recently attracted attention as a potential biological control agent against *Passiflora foetida*, an invasive vine that causes significant ecological and economic damage in Australia (Clavijo-Giraldo et al., 2025. *Unpublished*). Although a considerable number of cryptorhynchine weevils are known as agricultural pests, some species are being studied for their potential as biological control agents. *P. inermis* is one such example, exhibiting high host specificity towards *P. foetida* and inducing gall formation that weakens or kills the host plant. However, the lack of molecular data for *Philonis* hampers its accurate phylogenetic placement and limits the development of molecular tools for its identification and monitoring in both native and introduced ranges.

Mitochondrial genomes or mitogenomes have become fundamental tools for understanding the evolutionary history, systematics, and ecology of insects. ^1, 2^ In Coleoptera, the increasing availability of complete mitogenomes has facilitated robust phylogenetic analyses at various taxonomic levels, revealing patterns of diversification and adaptation in highly speciose families such as Curculionidae and other related weevil lineages. ^3– 5^ Despite these advances, mitogenomic data remain scarce for the subfamily Cryptorhynchinae, which encompasses a highly diverse assemblage of weevils with complex host-plant interactions.

Although mitogenomes are powerful markers, their use in phylogenetic studies is not without limitations. ^6, 7^ High substitution rates—particularly in third codon positions—can lead to substitution saturation in deep evolutionary branches, reducing the ability to accurately recover relationships among distantly related taxa. Such constraints are less problematic at shallow timescales, where mitochondrial genomes retain strong resolving power for population-level analyses and recent divergences. Because the present study focuses on intraspecific and closely related lineages, mitogenomic data remain well suited to our research objectives. ^8^

Recent mitogenomic studies within Cryptorhynchinae have mainly focused on species of economic concern, particularly pests of woody plants and crops. Examples include *Eucryptorhynchus chinensis* and *E. brandti*, pests of *Ailanthus altissima* in China, ^9, 10^ and the mango seed weevils *Sternochetus gravis*, *S. mangiferae*, and *S. olivieri.* ^11^ The hyperdiverse genus *Trigonopterus*, with hundreds of recently described species, ^12^ also provides important comparative resources. Although related genera such as *Aclees* ^13^ are not part of Cryptorhynchinae, they remain relevant for broader curculionid comparisons. In parallel with mitogenomic research, the use of the mitochondrial *Cytochrome c* oxidase subunit I (COI) gene as a DNA barcode has become an essential tool for species identification, biodiversity assessment, and the detection of cryptic diversity in weevils and other insects. ^14, 15^ Despite its broad application in Curculionidae, COI barcoding data have been scarce for Neotropical Cryptorhynchinae, limiting our understanding of species boundaries and population structure in this group. Until now, no complete mitogenomes have been available for any Neotropical representative of Cryptorhynchinae, making *P. inermis* the first of its kind. The integration of mitogenomic and COI barcode data presented here provides a valuable reference for future studies on species delimitation, comparative genomics, and the development of molecular tools for monitoring and management of potential biological control agents.

In this context, the accurate delimitation of *P. inermis* is critical for any potential classical biological control strategy against *P. foetida.* Correct species identification, coupled with low intraspecific genetic variability, ensures the reliability and safety of introducing candidate agents into new environments. By combining complete mitochondrial genome sequencing with COI barcode analysis, this study not only provides the first comprehensive molecular characterization of *P. inermis*, but also demonstrates the utility of COI as a diagnostic marker for its unambiguous identification. These resources will serve as a foundation for both fundamental studies on Cryptorhynchinae evolution and applied research aimed at evaluating *P. inermis* as a safe and effective biocontrol agent.

This study aims to generate the first low-coverage genome sequencing–based mitochondrial genome of *Philonis inermis* and to integrate these data with COI barcode sequences in order to refine molecular identification and enable comparative analyses within Cryptorhynchinae. Through comparative analyses with available mitogenomes of related genera ( *Eucryptorhynchus*, *Sternochetus*, and *Trigonopterus*), we explore patterns of gene arrangement, nucleotide composition, codon usage, and control region variability. These results not only fill a significant gap in the mitogenomic data of Neotropical Cryptorhynchinae but also provide essential molecular resources for evaluating *P. inermis* as a candidate biological control agent of *P. foetida*, linking fundamental evolutionary insights with applied biocontrol strategies.

## 2. Materials and methods

### 2.1 Sample collection and identification

Field surveys were conducted between 2021 and 2024 across dry forest habitats of northern Colombia (departments of Antioquia, Córdoba, and Bolívar; 0–200 m a.s.l.) to locate populations of *P. foetida* (Passifloraceae) exhibiting stem galls induced by *P. inermis.* The host plant was identified by botanist Wilder Buitrago Arbeláez (Herbarium of the Universidad de Antioquia, HUA, Medellín, Colombia) based on vegetative and floral characters following the diagnostic treatment reported elsewhere. ^16^ A voucher specimen of *P. foetida* was deposited at the HUA Herbarium under collection number HUA-1633.

Gall-bearing stems were excised using sterile scissors, placed in individual 50 mL sterile polypropylene tubes (Falcon, Cat. No. FALC-352070X25), and transported to the Entomology Laboratory of Universidad Nacional de Colombia (Medellín). Each gall was incubated separately under controlled environmental conditions (25 ± 2°C, 70 ± 5% relative humidity, 12:12 h light: dark cycle) until adult emergence to minimize contamination and sample mixing.

Adults were either preserved in 96% ethanol (Merck, Cat. No. 100983) for molecular work or mounted as pinned specimens for morphological examination. Species identification was confirmed through external morphological traits (rostrum length and curvature, elytral scale pattern, and sexual dimorphism) and dissection of male genitalia using a Leica EZ4 HD stereomicroscope (Leica Microsystems, Germany). Identification followed the diagnostic criteria of O’Brien ^17^ and comparisons with authenticated reference material. Voucher specimens were deposited in the Francisco Luis Gallego Entomological Museum (Universidad Nacional de Colombia, Medellín) under catalog numbers NC 65188–NC 65220.

### 2.2 DNA extraction, library preparation, and sequencing

Genomic DNA was extracted from approximately 25 mg of thoracic muscle from ethanol-preserved adults using the DNeasy Blood & Tissue Kit (Qiagen, Germany; Cat. No. 69504) according to the manufacturer’s protocol. Each extraction used 180 μL Buffer ATL and 20 μL Proteinase K, with overnight digestion at 56°C, followed by standard purification and elution in 100 μL Buffer AE.

DNA concentration and purity were quantified using a NanoDrop 2000/2000c spectrophotometer (Thermo Fisher Scientific, USA; Cat. No. ND-2000) and verified by 1% agarose gel electrophoresis in 1× TAE buffer (Invitrogen, USA; Cat. No. 15558-042) with GelRed stain (Biotium, USA; Cat. No. 41003). Only samples with concentrations ≥1 ng μL ^−1^ and 260/280 and 230/260 ratios between 1.8 and 2.0 were used for sequencing.

Two high-quality DNA extracts, designated Pinermis_Ant (Antioquia) and Pinermis_Cor (Córdoba), were selected for shallow whole-genome sequencing (~7× coverage). Paired-end libraries (2 × 150 bp; ~350 bp insert size) were prepared using the NEBNext Ultra II DNA Library Prep Kit for Illumina (New England Biolabs, USA; Cat. No. E7645L) following the manufacturer’s protocol, including size selection with AMPure XP magnetic beads (Beckman Coulter, USA; Cat. No. A63881). Library concentrations were measured using a Qubit 4 Fluorometer (Invitrogen, USA; Cat. No. Q33226) and the Qubit dsDNA HS Assay Kit (Cat. No. Q32854). Libraries were pooled equimolarly and sequenced on an Illumina NovaSeq X platform (Illumina, USA) at Macrogen Inc. (Seoul, South Korea), generating approximately 8–10 Gb of raw paired-end sequencing data per sample, corresponding to an estimated genomic coverage of ~7×.

### 2.3 COI amplification and DNA barcoding

The COI barcode region (cox1) was amplified using standard insect primers LCO1490 and HCO2198. ^18^ PCR amplifications were carried out in 50 μL reactions containing 4 μL of genomic DNA template, 0.25 U μL ^−1^ of Taq DNA Polymerase (Thermo Fisher Scientific, USA; Cat. No. EP0402), 5 μL of 10× PCR reaction buffer (supplied with the enzyme), 1 μL of 10 mM dNTP mix (Invitrogen, USA; Cat. No. 18427-013), and 1.5 μL each of forward and reverse primers (10 μM), with the remaining volume adjusted to 50 μL using nuclease-free water (Thermo Fisher Scientific, Cat. No. AM9937). Thermal cycling was performed in a T100 Bio-Rad 96-Well Thermal Cycler (Bio-Rad Laboratories, Inc., USA) under the following conditions: initial denaturation at 95°C for 5 min; 45 cycles of denaturation at 95°C for 40 s, primer annealing at 51°C for 60 s, and extension at 72°C for 45 s; followed by a final elongation step at 72°C for 10 min. PCR products were purified and sequenced by Macrogen Inc. (Seoul, Korea).

A total of 20 COI sequences from *P. inermis* were analyzed together with 24 additional COI sequences from American Cryptorhynchinae species retrieved from GenBank. Sequence alignment was conducted in MAFFT v7.525, ^19^ and pairwise genetic distances were calculated under the Kimura 2-Parameter (K2P) model using the ape v5.8 ^20^ package in R. All COI sequences were additionally uploaded to the Barcode of Life Data System (BOLD), where they were linked to vouchered specimens with associated images and evaluated using BOLD workbench tools. ^21^ Intraspecific K2P distances were estimated only for species with more than four available COI sequences, providing an approximate measure of within-species variation in the barcode region. A Maximum likelihood (ML) phylogenetic tree based on COI sequences was conducted using the extended model selection followed by tree inference and ultra-fast non-parametric bootstrap with 1,000 replicates to evaluate node support in IQ-Tree v2.0.3. ^22^

### 2.4 Read processing, genome assembly, and gene ortholog assessment

Raw Illumina paired-end reads were quality-filtered and trimmed using fastp, ^23^ and the resulting high-quality reads were assembled de novo with SPAdes. ^24^ Potential exogenous contamination was evaluated by classifying quality-filtered reads from both *P. inermis* libraries (Pinermis_Ant and Pinermis_Cor) with Kraken2 (v2.1.2) using the PlusPF reference database. ^25^ Kraken2 reports were then used to estimate the relative contribution of major taxonomic groups, including Metazoa, Bacteria, Fungi, Viruses, and unclassified reads.

We evaluated the completeness of the assembly using Benchmarking Universal Single Copy Orthologs (BUSCO v. 6.0) ^26^ for both sequenced individuals (Antioquia and Córdoba samples) against endopterygota_odb12 database. Complete single-copy genes were extracted from both *P. inermis* (Antioquia and Córdoba) samples and annotated by Clusters of Orthologous Genes (COG) using eggNOG-mapper ( http://eggnog-mapper.embl.de/). In addition, duplicated BUSCOs and multicopy nuclear markers—including rRNA and histone loci—were recovered to provide supplementary phylogenomic resources.

### 2.5 Mitogenome annotation and phylogenetic tree

Assembled contigs were screened for mitochondrial sequences by BLASTN comparison to reference mitogenomes from Cryptorhynchinae ( *Eucryptorhynchus brandti* - NC_025945.1; *E. chinensis* - NC_026719.1; *Trigonopterus selaruensis* - NC_050886.1; *T. tanimbarensis* - NC_050887.1; *T. jasminae* - NC_050888.1; *T. triradiatus* - NC_050889.1; *T. singkawangensis* - NC_050890.1; *T. carinirostris* - NC_050891.1; *T. kotamobagensis* - NC_050892.1; *T. porg* - NC_050893.1; *Sternochetus mangiferae* - NC_068213.1; *S. gravis* - NC_068212.1; *S. olivieri* - NC_068214.1). Mitochondrial genomes annotation was performed using GeSeq and OGDRAW webserver. ^27^ The mitogenome organization was compared using the GLOBAL multi-GFF3 output retrieved from the OGDRAW webserver, ^27^ and subsequently processed with a custom R script.

Mitogenome sequences were aligned using MAFFT v7.525 ^19^ under the auto strategy. Poorly aligned regions were inspected and trimmed where necessary. Maximum likelihood (ML) phylogenetic inference was performed using IQ-TREE v2.0.3 ^22^ with automatic model selection (ModelFinder) and node support assessed with 1,000 ultrafast bootstrap replicates. The resulting tree was visualized and edited in R.

## 3. Results

### 3.1 Genome skimming of *P. inermis*

We obtained a total of 23.5 and 15.6 million reads for the *P. inermis* individuals from Antioquia and Córdoba, respectively ( Table 1). The assemblies yielded N50 values of 21 and 50, with approximately 213,000 and 233,000 contigs for the Antioquia and Córdoba individuals, respectively ( Table 1). Taxonomic profiling of unmapped reads using Kraken2 (confidence = 0.5) indicated low levels of microbial contamination in both libraries. Bacterial reads accounted for ~8% of unmapped reads in both samples, while fungal and viral reads were negligible (<0.3% and <0.01%, respectively). A proportion of unmapped reads (11.0% in *Pinermis_Ant* and 17.8% in *Pinermis_Cor*) was assigned to *Homo sapiens*; however, these reads did not assemble into contigs and were excluded from downstream analyses. Overall, contamination was minor and did not impact assembly quality or mitogenome reconstruction.

**Table 1.** Genome sequencing, assembly, and completeness statistics for two individuals of *Philonis inermis.*

Parameter	Pinermis_Ant	Pinermis_Cor
High-quality reads	23 495 598	15 632 458
Assembled genome size (Mbp)	263.1	139.3
Contigs N50 (Kbp)	21	50
Contigs number	213 631	233 401
Complete BUSCOs	1954	201
Complete and single-copy BUSCOs	1930	196
Complete and duplicated BUSCOs	24	5

**Figure 1.**
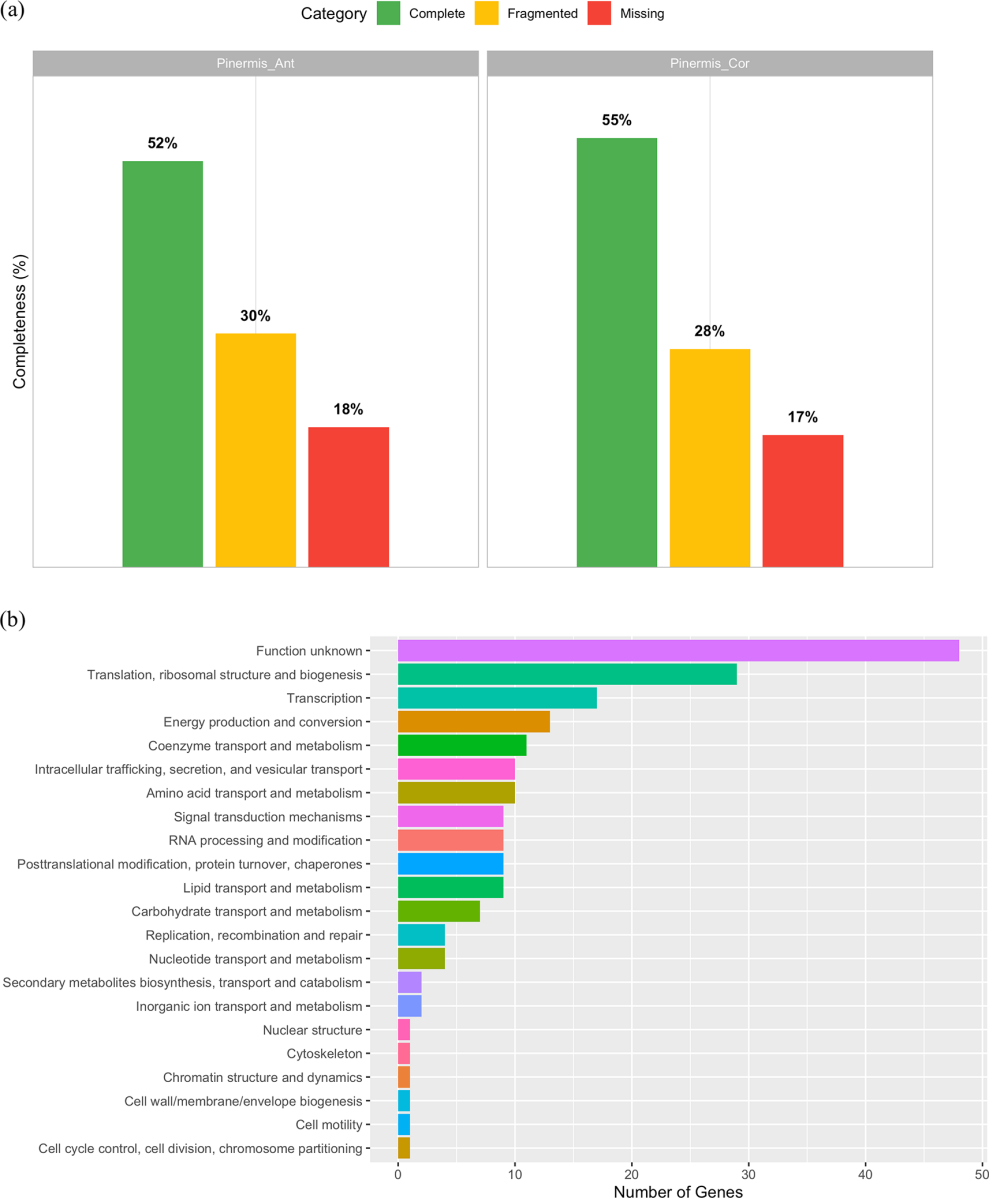
Genome-skimming assessment for *Philonis inermis.* (a) BUSCO completeness profiles for two genome assemblies (Endopterygota_odb12; 3,754 genes): Pinermis_Ant (52% complete, 30% fragmented) and Pinermis_Cor (55% complete, 28% fragmented). (b) Distribution of COG functional categories for the 196 single-copy BUSCO orthologs recovered in both assemblies.

BUSCO analysis of the shallow-genome assemblies recovered, out of 3,754 expected Endopterygota genes, 52% complete and 30% fragmented in Pinermis_Ant, and 55% complete and 28% fragmented in Pinermis_Cor ( Figure 1a), yielding >45% non-complete (fragmented + missing) loci in both assemblies, consistent with notable fragmentation. Despite this, we identified 196 single-copy orthologs having non-stop codons shared by both samples that were assigned to 22 COG functional categories (Supplementary S1). The distribution was dominated by Function unknown (24.1%), followed by Translation, ribosomal structure and biogenesis (14.6%) and Transcription (8.5%) ( Figure 1b). Core cellular and metabolic processes were moderately represented, including Energy production and conversion (6.5%), Coenzyme transport and metabolism (5.5%), Amino acid transport and metabolism (5.0%), Intracellular trafficking, secretion, and vesicular transport (5.0%), and RNA processing; lipid metabolism; post-translational modification/chaperones; signal transduction each at ~4.5%. Carbohydrate metabolism reached 3.5%, whereas nucleotide metabolism and replication/repair were ~2.0% ( Figure 1b). Overall, even with fragmented assemblies, conserved informational functions are well captured, while a substantial fraction of single-copy orthologs remains uncharacterized. In addition to these single-copy loci, we also recovered 28 duplicated BUSCOs spanning diverse nuclear functions (including amino-acid and carbohydrate metabolism, redox processes, transport, and chromatin modification), as well as a broad set of multicopy rRNA genes (18S, 28S, 16S) and more than 150 histone-related loci (canonical H2A/H2B/H3/H4, histone variants, and associated chromatin-modifying proteins) from both assemblies (Supplementary Tables S2–S3). These multicopy nuclear markers provide an additional reservoir of phylogenetically informative sequences for future comparative studies.

### 3.2 Mitogenome characterization of *Philonis inermis*

**Figure 2.**
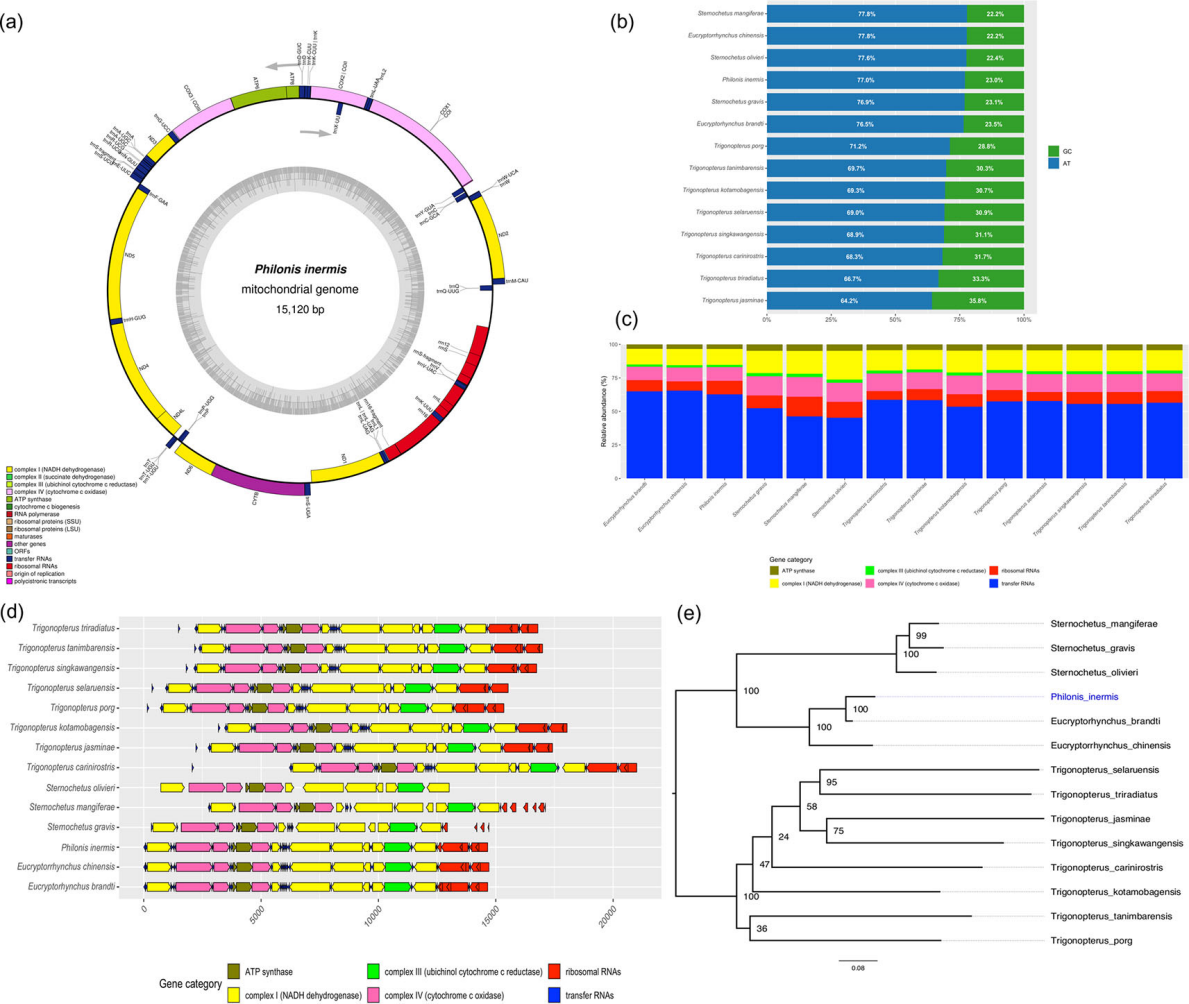
Mitogenome characterization of *Philonis inermis.* (a) Circular map of the complete mitochondrial genome of *P. inermis* showing gene annotation and organization. (b) Comparative nucleotide composition (AT vs. GC content) of *P. inermis* and other Cryptorhynchinae mitogenomes. (c) Relative abundance of annotated gene categories across *P. inermis* and related Cryptorhynchinae species. (d) Synteny and structural arrangement of annotated genes in *P. inermis* compared with related Cryptorhynchinae mitogenomes. (e) Maximum likelihood phylogenetic tree inferred from complete mitogenome sequences of *P. inermis* and representative Cryptorhynchinae species. GenBank accession numbers for all included sequences are provided in the Methods section. Bootstrap support values (1,000 replicates) are shown at nodes.

The representative mitochondrial genome of *P. inermis* was 15,120 bp in length and contained 36 features typically found in insect mitogenomes: 13 protein-coding genes (PCGs), two ribosomal RNAs (rRNAs), and 21 transfer RNAs (tRNAs). The only gene not identified was tRNA-Ile (trnI) ( Figure 2a). The overall nucleotide composition was strongly biased toward adenine and thymine (A+T = 77.02%), a pattern widely reported in mitochondrial genomes of weevils and other insect and arthropod taxa ( Figure 2b).

Comparative analyses revealed that *Eucryptorhynchus* spp. and *P. inermis* possess the highest total gene counts among the examined Cryptorhynchinae, primarily due to an increased number of tRNAs ( Figure 2c). In contrast, *Sternochetus* spp. exhibit a lower overall gene count, reflecting a reduction in tRNA genes. The numbers of protein-coding genes, rRNAs, and other categories remain largely conserved across genera, with only minor differences observed ( Figure 2c). Furthremore the mitochondrial genome structure of *P. inermis* was highly conserved and syntenic with other Cryptorhynchinae species, without major rearrangements, despite minor variations in gene spacing and orientation ( Figure 2d). These patterns indicate strong conservation of mitochondrial gene content and organization within Cryptorhynchinae, with variation mainly associated with tRNA gene numbers. The preliminary phylogenetic reconstruction based on complete mitochondrial genome sequences placed *P. inermis* as sister to the *Eucryptorrhynchus* clade with strong support (BS = 100), while *Sternochetus* species clustered into a distinct lineage within Cryptorhynchinae. In contrast, relationships among *Trigonopterus* species were less resolved, with several nodes showing low support ( Figure 2e). These results should be considered preliminary, as the current topology is influenced by the limited and uneven mitogenomic representation available in public databases.

### 3.3 DNA barcoding and intraspecific variation of COI gene in *P. inermis*

**Figure 3.**
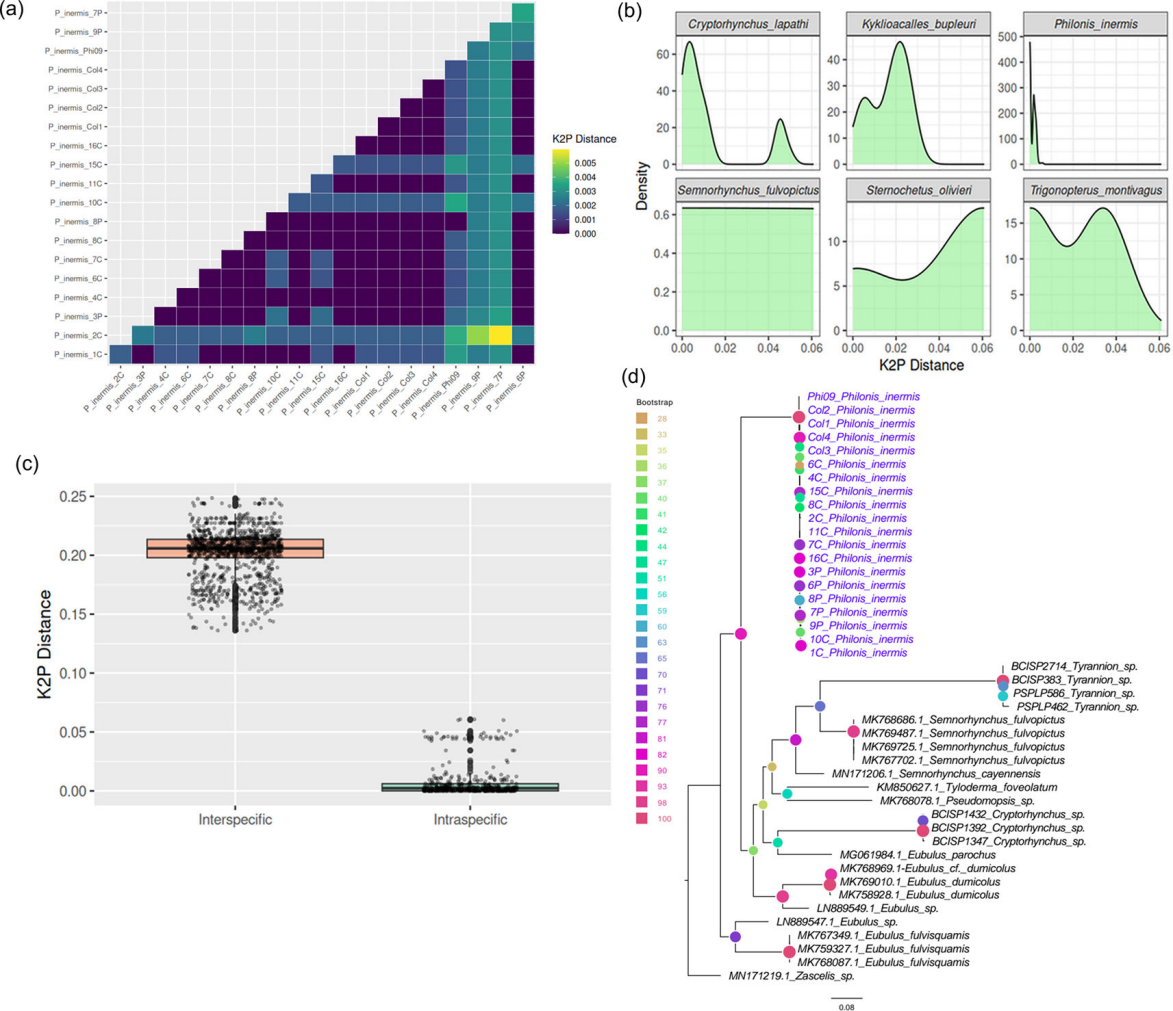
COI barcode variation and phylogenetic placement of *Philonis inermis.* (a) Pairwise Kimura 2-Parameter (K2P) distances among Colombian *P. inermis* specimens. (b) Density plots of K2P distances in *P. inermis* compared with selected Cryptorhynchinae species. (c) Comparison of intra- and interspecific K2P distances across Cryptorhynchinae, illustrating a clear barcode gap. (d) Maximum likelihood tree based on COI sequences showing *P. inermis* as a distinct, well-supported clade among Neotropical Cryptorhynchinae.

The COI barcode sequences of *P. inermis* from multiple Colombian populations exhibited extremely low intraspecific divergence, with pairwise Kimura 2-Parameter (K2P) distances ranging from 0 to 0.006 ( Figure 3a), consistent across external and BOLD-based analyses. This low genetic variability, evident from both the distance matrix and density distributions, supports the genetic homogeneity of *P. inermis* across sampled localities, especially when compared with other Cryptorhynchinae species ( Figure 3b). Comparison with additional Cryptorhynchinae COI sequences from GenBank revealed a clear barcode gap: intraspecific K2P distances in *P. inermis* were substantially lower than interspecific distances across Cryptorhynchinae, which were typically greater than 0.15 ( Figure 3c). This distinct separation highlights the effectiveness of COI barcoding for reliably identifying *P. inermis* and distinguishing it from related taxa. A maximum likelihood tree constructed from COI sequences ( Figure 3d) clustered all *P. inermis* specimens together with strong bootstrap support, forming a well-defined lineage among Neotropical Cryptorhynchinae and further validating its molecular distinctiveness.

## 4. Discussion

Despite family Curculionoidae is rendering as one of the most diverse groups of Coleoptera, encompasses approximately 62,000 species distributed among 5,800 described genera, ^28^ many attributes about genome structure, diversity, and ecological role of Neotropical species belonging to Crypthorynchinae subfamily such *P. inermis* is poorly unknowledge so far.

The weevils of the Neotropical genus *Philonis* (Curculionidae: Cryptorhynchinae) represent a largely unexplored lineage within the diverse assemblage of gall-inducing insects. Here, we present the first genomic resources for this genus based on low-coverage genome sequencing, including the complete mitochondrial genome and an assessment of COI DNA barcode accuracy. These data provide a foundation for future studies of genetic diversity, population structure, and evolutionary history, and may also inform applied research in biological control of the invasive vine *Passiflora foetida* L.

The relatively fragmented assemblies reflect the shallow genome-skimming strategy adopted in this study, which was specifically designed to recover the complete mitochondrial genome and representative nuclear markers rather than to generate a high-contiguity nuclear assembly. Our primary objective was to establish genomic resources for taxonomic validation and phylogenetic inference, for which modest sequencing depth is sufficient. Although increased coverage and the incorporation of long-read technologies would substantially improve assembly contiguity, such approaches were beyond the scope of the present study.

Consistent with the limited coverage, BUSCO profiles indicate that neither assembly captures the complete nuclear gene space; nonetheless, we recovered 196 shared single-copy orthologs, 191 of which contain intact open reading frames and were assigned to 22 COG functional categories. These loci encompass core informational and metabolic functions and collectively constitute a robust marker panel for future comparative and phylogenomic analyses in *Philonis inermis* and related Neotropical Cryptorhynchinae. The substantial proportion of unclassified COGs further highlights the limited genomic characterization of this lineage and underscores the potential for discovery as additional genomic data become available. In parallel, the recovery of duplicated BUSCOs and extensive rRNA and histone gene clusters provides complementary multicopy markers that may prove useful for resolving deeper or more complex evolutionary relationships. Future work integrating long-read sequencing and Hi-C scaffolding will be essential to resolve repetitive regions, reduce fragmentation, and ultimately achieve chromosome-scale assemblies for this ecologically important genus.

The mitochondrial genome of *P. inermis* exhibits a nucleotide composition biased toward (A+T), as well as gene order and orientation that are highly conserved, and consistent with the ancestral insect mitogenome architecture. Comparative analyses with complete mitogenomes from other Cryptorhynchinae genera revealed strong structural and syntenic conservation across the group. ^9, 10, 12, 29^ As reported for other species such as *Eucryptorrhynchus chinensis* and *E. brandti* the mitogenome of *P. inermis* lacks an identifiable deficiency of tRNA-Ile gene. ^10^ The trnI gene is likely located within the highly variable control region, where high elevated divergence hampers automated annotation and precise boundary delimitation, making its detection a common challenge. ^30^

Mitogenome-based tree place *Philonis* close to *Eucryptorrhynchus*, but we treat this as preliminary, given the limited and uneven mitogenomic sampling across Cryptorhynchinae. Broad, multi-gene frameworks show that relationships above the genus level can be difficult to stabilize in this subfamily, and that extensive sampling across loci is required to resolve deeper nodes. ^31, 32^ In particular, Riedel et al. ^32^ recovered a large-scale molecular phylogeny that points to an American origin for Cryptorhynchinae and highlights the value of integrating mitochondrial and nuclear markers for robust placement. Our results align with this perspective: the topology we report is a useful working hypothesis that should be tested with expanded taxon and locus coverage.

If proximity to *Eucryptorrhynchus* is confirmed, the comparison is ecologically instructive. *Ailanthus altissima* (Simaroubaceae), the tree-of-heaven, is a fast-growing invasive tree native to China that readily colonizes disturbed habitats and can displace native vegetation. Within this host context, *E. scrobiculatus* and *E. brandti* are highly specialized on *A. altissima.* In parts of their native range, they are considered pests; however, they have also been evaluated as potential biological control agents where *A. altissima* is invasive, and climate-suitability assessments suggest differential responses of the two congeners to future climates. ^10, 33^ This dual “pest vs. biocontrol” context underscores why clear systematics and robust diagnostics matter when considering host-specific herbivores for applied programs.

Interestingly, all other cryptorhynchine weevils with complete mitochondrial genomes currently reported are documented agricultural pests of economic importance in Asia or Oceania, highlighting a significant gap for Neotropical species. In contrast, *P. inermis* is a stem-galling specialist with a narrow host range restricted to *P. foetida*, an invasive vine in Australia. ^34^

At a broader scale, historical biogeography indicates a complex macroevolutionary backdrop for Cryptorhynchinae, with major diversity centers in the Neotropics and Australasia and signals consistent with an American origin. ^3, 32, 35^ Comparative insights from flightless *Trigonopterus* show repeated crossings of major biogeographic barriers (e.g., Wallace’s Line), rapid radiations and finely structured endemism, and these features complicate deep-time reconstructions when sampling is sparse. ^36^ These patterns provide a cautionary frame for interpreting single-marker or low-taxon trees and reinforce the need for expanded, balanced sampling when refining the placement of *Philonis.*

Assessment for COI gene DNA barcoding regions indicates high accuracy on molecular identification of *P. inermis*, with minimal genetic divergence among Colombian individuals from three populations (K2P distances ≤ 0.006). This low intraspecific variability underscores the genetic coherence of the species and confirms that the sampled populations across Colombia belong to the same taxonomic unit. Despite the limited flight ability of *P. inermis*, this genetic homogeneity may be influenced by passive dispersal associated with its host ( *P. foetida*), a hypothesis that warrants further investigation. Furthermore, both inter – and intraspecific genetic distance gaps support the COI barcode as a suitable tool for species identification in Neotropical cryptorhynchine weevils, exceeding values reported for related species from Asia, Europe, and Oceania. ^36, 37^ Under this scenario, our COI results supports unambiguous diagnosis of *P. inermis.* This pattern aligns with the central rationale for DNA barcoding as a standardized, taxonomically integrative tool. ^15, 38^ The maximum likelihood tree constructed from COI sequences clustered all *P. inermis* specimens together with strong statistical support, further confirming its genetic uniformity and separation from other Neotropical taxa. Collectively, these barcode results provide a robust molecular baseline for species identification and support the consideration of *P. inermis* as a promising candidate for biological control programs targeting *P. foetida.*

From an applied perspective, the combination the mitogenome and COI barcoding supports accurate recognition across Colombian populations, which is a prerequisite for any subsequent risk assessment. Furthermore, this approach strengthens integrative taxonomy, biosecurity, and classical weed biocontrol workflows. Barcodes offer interoperable, scalable diagnostics for look-alike taxa and immature stages, facilitate data sharing across laboratories and jurisdictions, and support post-release monitoring, both of which are key steps to minimize non-target risk. ^15, 38, 39^ The global invasive potential documented for *S. mangiferae* under climate change further illustrates why strong diagnostics and early detection pipelines are increasingly critical for curculionid lineages with agricultural relevance. ^40^

Limitations and next steps follow directly from our results. First, phylogenetic inferences from current mitogenome sampling should be treated as provisional. Resolving deeper nodes will require denser Neotropical sampling, including close relatives of *Philonis*, and multi-locus or genomic matrices that integrate nuclear markers. Second, expanding the geographic and host-associated sampling for COI (and additional markers) will help quantify population structure and confirm the breadth of the barcode gap. Third, low-coverage, short-read genome skimming inherently underrepresents repetitive and GC-extreme regions and can collapse recent paralogs, yielding fragmented assemblies and biasing functional annotations toward conserved single-copy genes; increasing sequencing depth and integrating long reads and Hi-C will mitigate these biases. Together, these steps will strengthen both the systematic placement of *Philonis* and the applied utility of its genetic resources for monitoring and potential biocontrol assessment.

## 5. Conclusions

We provide the first mitogenomic reference for *P. inermis* (15,120 bp; ~77% A+T), with conserved gene order and deficiency of tRNA-Ile likely obscured within the variable control region. Maximum-likelihood analyses recover *P. inermis* as a well-supported clade and tentatively near *Eucryptorrhynchus*, a hypothesis that awaits denser taxon and locus sampling. COI barcodes from 20 Colombian individuals show extremely low intraspecific divergence (K2P ≤ 0.006) and a pronounced barcode gap from other American cryptorhynchine weevils, enabling reliable, field-ready diagnostics. Low-coverage genome sequencing recovered 196 single-copy orthologs along with a complementary set of duplicated BUSCOs and multicopy rRNA and histone genes, furnishing anchors for future genome-scale phylogenetics and comparative genomics. Together, these resources demonstrate the utility of COI barcoding for the current molecular identification of *P. inermis*, establish a molecular foundation for systematics and population-level studies, and inform the risk-aware evaluation of *P. inermis* as a host-specific candidate for classical biological control of *P. foetida*. Priority next steps include long-read and Hi-C genome assemblies and expanded geographic and taxonomic sampling—including nuclear markers—to resolve deeper relationships and better quantify population structure.

## Data Availability

The raw sequencing datasets generated in this study have been deposited in the NCBI Sequence Read Archive (SRA) under BioProject accession number PRJNA1322535. The complete mitochondrial genome and COI partial sequences of *Philonis inermis* are available in GenBank under accession numbers PX645216, and PX353910–PX353929. The corresponding barcode dataset, including voucher information and specimen images, is available in the Barcode of Life Data System (BOLD) under PINEBIN: AHI5100. **Figshare/Zenodo Repository** “ *Supplementary Tables S1–S3 – Characterization of the Complete Mitochondrial Genome and Evaluation of COI Barcoding in Philonis inermis (Coleoptera: Curculionidae: Cryptorhynchinae) Using Genome Skimming.*” https://doi.org/10.5281/zenodo.17793370. This project contains the following extended data:
•
**Supplementary Table S1. **Single-copy BUSCO orthologs recovered from *Philonis inermis* genome skimming assemblies, including locus identifiers, functional annotations, and sequence lengths. •
**Supplementary Table S2. **List of duplicated (multicopy) BUSCO genes detected in the *endopterygota_odb12* run, together with annotations for each multicopy ortholog. These loci represent additional nuclear genes of potential interest for phylogenomic studies. •
**Supplementary Table S3. **Extracted multicopy nuclear markers—including complete or partial **rRNA clusters** and **core histone genes** recovered directly from the *P. inermis* assemblies. Only loci ≥1 kb were retained. For each locus, we provide coordinates, strand orientation, percent identity, alignment length, and extracted sequence. • All extended data files are publicly available at Zenodo (DOI: 10.5281/zenodo.17793370). Data are available under the terms of the Creative Commons Attribution 4.0 International license (CC-BY 4.0).

## References

[1] Cameron SL : Insect Mitochondrial Genomics: Implications for Evolution and Phylogeny. *Annu. Rev. Entomol.* 2014;59:95–117. 10.1146/annurev-ento-011613-162007 24160435

[2] Timmermans MJTN Barton C Haran J : Family-Level Sampling of Mitochondrial Genomes in Coleoptera: Compositional Heterogeneity and Phylogenetics. *Genome Biol. Evol.* 2016;8:161–175. 10.1093/gbe/evv241 26645679 PMC4758238

[3] Letsch H Vukotić S Gottsberger B : The Phylogeny of Ceutorhynchine Weevils (Ceutorhynchinae, Curculionidae): Mitogenome Data Improve the Resolution of Tribal Relationships. *Syst. Entomol.* 2024;49:624–634. 10.1111/syen.12635

[4] Song N Li X Yin X : The Mitochondrial Genome of *Apion Squamigerum* (Coleoptera, Curculionoidea, Brentidae) and the Phylogenetic Implications. *PeerJ.* 2020;8:e8386. 10.7717/peerj.8386 31976182 PMC6964704

[5] Ren J Zhang R : Delimiting Species, Revealing Cryptic Diversity in Molytinae (Coleoptera: Curculionidae) Weevil through DNA Barcoding. *J. Insect Sci.* 2024;24:25. 10.1093/jisesa/ieae083 39348593 PMC11441576

[6] Xia X Xie Z Salemi M : An index of substitution saturation and its application. *Mol Phylogenet Evol.* 2003;26:1–7. 10.1016/S1055-7903(02)00326-3 12470932

[7] Hassanin A Léger N Deutsch J : Evidence for Multiple Reversals of Asymmetric Mutational Constraints during the Evolution of the Mitochondrial Genome of Metazoa, and Consequences for Phylogenetic Inferences. *Syst Biol.* 2005;54:277–298. 10.1080/10635150590947843 16021696

[8] Phillips MJ Penny D : The root of the mammalian tree inferred from whole mitochondrial genomes. *Mol Phylo and Evol.* 2003;28:171–185. 10.1016/S1055-7903(03)00057-5 12878457

[9] Nan X Wei C He H : The Complete Mitogenome of *Eucryptorrhynchus Brandti* (Harold) (Insecta: Coleoptera: Curculionidae). *Mitochondrial DNA Part A.* 2016;27:2060–2061. 10.3109/19401736.2014.982556 25427809

[10] Liu Z-K Gao P Ashraf MA : The Complete Mitochondrial Genomes of Two Weevils, Eucryptorrhynchus Chinensis and E. Brandti: Conserved Genome Arrangement in Curculionidae and Deficiency of tRNA-Ile Gene. *Open Life Sci.* 2016;11:458–469. 10.1515/biol-2016-0060

[11] Zhang L Li Y Ge X : Mitochondrial Genomes of *Sternochetus* Species (Coleoptera: Curculionidae) and the Phylogenetic Implications. *Arch. Insect Biochem. Physiol.* 2022;111:e21898. 10.1002/arch.21898 35434835

[12] Narakusumo RP Riedel A Pons J : Mitochondrial Genomes of Twelve Species of Hyperdiverse *Trigonopterus* Weevils. *PeerJ.* 2020;8:e10017. 10.7717/peerj.10017 33083123 PMC7566755

[13] Schütte A Stüben P Astrin J : Molecular Weevil Identification Project: A Thoroughly Curated Barcode Release of 1300 Western Palearctic Weevil Species (Coleoptera, Curculionoidea). *BDJ.* 2023;11:e96438. 10.3897/BDJ.11.e96438 38357418 PMC10865102

[14] Hong K-J Ki W Lee I-J : The Complete Mitochondrial Genome of *Aclees Taiwanensis* Kôno, 1933 (Coleoptera: Curculionidae). *Mitochondrial DNA Part B.* 2022;7:1460–1462. 10.1080/23802359.2022.2107440 35979394 PMC9377266

[15] Hajibabaei M Singer GAC Hebert PDN : DNA Barcoding: How It Complements Taxonomy, Molecular Phylogenetics and Population Genetics. *Trends Genet.* 2007;23:167–172. 10.1016/j.tig.2007.02.001 17316886

[16] Vanderplank J : A Revision of Passiflora Section Dysosmia: Passifloraceae. *Curtis's Bot. Mag.* 2013 Dec [cited 2025 Oct 7];30(4):318–387. 10.1111/curt.12050

[17] O’Brien CW : Revision of the Neotropical weevil genus Philonis (Cryptorhynchinae: Curculionidae: Coleoptera). *Southwest. Entomol.* 1984;9:232–239.

[18] Folmer O Black M Hoeh W : DNA Primers for Amplification of Mitochondrial Cytochrome c Oxidase Subunit I from Diverse Metazoan Invertebrates. *Mol. Mar. Biol. Biotechnol.* 1994;3:294–299. 7881515

[19] Katoh K Standley DM : MAFFT Multiple Sequence Alignment Software Version 7: Improvements in Performance and Usability. *Mol. Biol. Evol.* 2013;30:772–780. 10.1093/molbev/mst010 23329690 PMC3603318

[20] Paradis E Schliep K : Ape 5.0: An Environment for Modern Phylogenetics and Evolutionary Analyses in R. *Bioinformatics.* 2019;35:526–528. 10.1093/bioinformatics/bty633 30016406

[21] Ratnasingham S Hebert PDN : A DNA-Based Registry for All Animal Species: The Barcode Index Number (BIN) System. *PLoS One.* 2013;8:e66213. 10.1371/journal.pone.0066213 23861743 PMC3704603

[22] Minh BQ Schmidt HA Chernomor O : IQ-TREE 2: New Models and Efficient Methods for Phylogenetic Inference in the Genomic Era. *Mol. Biol. Evol.* 2020;37:1530–1534. 10.1093/molbev/msaa015 32011700 PMC7182206

[23] Chen S Zhou Y Chen Y : Fastp: An Ultra-Fast All-in-One FASTQ Preprocessor. *Bioinformatics.* 2018;34:i884–i890. 10.1093/bioinformatics/bty560 30423086 PMC6129281

[24] Prjibelski A Antipov D Meleshko D : Using SPAdes De Novo Assembler. *Curr. Protoc. Bioinformatics.* 2020;70:e102. 10.1002/cpbi.102 32559359

[25] Wood DE Lu J Langmead B : Improved metagenomic analysis with Kraken 2. *Genome Biol.* 2019;20:257. 10.1186/s13059-019-1891-0 31779668 PMC6883579

[26] Tegenfeldt F Kuznetsov D Manni M : OrthoDB and BUSCO Update: Annotation of Orthologs with Wider Sampling of Genomes. *Nucleic Acids Res.* 2025;53:D516–D522. 10.1093/nar/gkae987 39535043 PMC11701741

[27] Tillich M Lehwark P Pellizzer T : GeSeq – Versatile and Accurate Annotation of Organelle Genomes. *Nucleic Acids Res.* 2017;45:W6–W11. 10.1093/nar/gkx391 28486635 PMC5570176

[28] Oberprieler RG Marvaldi AE Anderson RS : Weevils, Weevils, Weevils Everywhere. *Zootaxa.* 2007;1668. 10.11646/zootaxa.1668.1.24

[29] Li X Li R Rao F : Genetic Characterization of 2 *Ceutorhynchus* (Coleoptera: Curculionidae) Weevils with Mitogenomes and Insights into the Phylogeny and Evolution of Related Weevils. *J. Insect Sci.* 2024;24:14. 10.1093/jisesa/ieae038 38536151 PMC10972553

[30] Laslett D Canbäck B : ARWEN: A Program to Detect tRNA Genes in Metazoan Mitochondrial Nucleotide Sequences. *Bioinformatics.* 2008;24:172–175. 10.1093/bioinformatics/btm573 18033792

[31] Astrin JJ Stüben PE : Phylogeny in Cryptic Weevils: Molecules, Morphology and New Genera of Western Palaearctic Cryptorhynchinae (Coleoptera:Curculionidae). *Invert. Systematics.* 2008;22:503–522. 10.1071/IS07057

[32] Riedel A Tänzler R Pons J : Large-scale Molecular Phylogeny of Cryptorhynchinae (Coleoptera, Curculionidae) from Multiple Genes Suggests American Origin and Later Australian Radiation. *Syst. Entomol.* 2016;41:492–503. 10.1111/syen.12170

[33] Ding W Li H Wen J : Response of the Invasive Plant *Ailanthus Altissima* (Mill.) Swingle and Its Two Important Natural Enemies ( *Eucryptorrhynchus Scrobiculatus* (Motschulsky) and *E. Brandti* (Harold)) to Climate Change. *Ecol. Indic.* 2022;143:109408. 10.1016/j.ecolind.2022.109408

[34] Jucker T Long V Pozzari D : Developing Effective Management Solutions for Controlling Stinking Passionflower (Passiflora Foetida) and Promoting the Recovery of Native Biodiversity in Northern Australia. *Biol. Invasions.* 2020;22:2737–2748. 10.1007/s10530-020-02295-5

[35] Letsch H Balke M Toussaint EFA : Historical Biogeography of the Hyperdiverse Hidden Snout Weevils (Coleoptera, Curculionidae, Cryptorhynchinae). *Syst. Entomol.* 2020;45:312–326. 10.1111/syen.12396

[36] Tänzler R Van Dam MH Toussaint EFA : Macroevolution of Hyperdiverse Flightless Beetles Reflects the Complex Geological History of the Sunda Arc. *Sci. Rep.* 2016;6:18793. 10.1038/srep18793 26742575 PMC4732383

[37] Narakusumo RP Balke M Riedel A : Seven New Species of Trigonopterus Fauvel (Coleoptera, Curculionidae) from the Tanimbar Archipelago. *ZK.* 2019;888:75–93. 10.3897/zookeys.888.38642 31754320 PMC6861340

[38] Armstrong KF Ball SL : DNA Barcodes for Biosecurity: Invasive Species Identification. *Philos. Trans. R. Soc. B.* 2005;360:1813–1823. 10.1098/rstb.2005.1713 16214740 PMC1609225

[39] Gaskin JF Bon M-C Cock MJW : Applying Molecular-Based Approaches to Classical Biological Control of Weeds. *Biol. Control.* 2011;58:1–21. 10.1016/j.biocontrol.2011.03.015

[40] Aidoo OF Amaro GC Souza PGC : Climate Change Impacts on Worldwide Ecological Niche and Invasive Potential of *Sternochetus Mangiferae.* *Pest Manag. Sci.* 2025;81:667–677. 10.1002/ps.8465 39381897 PMC11716358

