# Supplementary material for: Characterization of the Complete Mitochondrial Genome and Evaluation of COI Barcoding in Philonis inermis (Coleoptera: Curculionidae: Cryptorhynchinae) Using Genome Skimming
Source: F1000Res. 2026 Apr 24;14:1174. Originally published 2025 Oct 28. [Version 3] doi: 10.12688/f1000research.170584.3 (PMC13062764; doi:10.12688/f1000research.170584.3)
Supplement: Peer review history

# Reviewer report: Reviewer response for version 2

**DOI:** 10.5256/f1000research.197074.r468386

**Published:** 2026-04-08

**Authors:** Renee Ali

**Affiliations:** 1 johns Hopkins Bloomberg School of Public Health, Baltimore, Maryland, USA

The authors have considered recommendations and made appropriate changes which has improved the manuscript making it suitable for indexing.

Is the work clearly and accurately presented and does it cite the current literature?

Partly

If applicable, is the statistical analysis and its interpretation appropriate?

Partly

Are all the source data underlying the results available to ensure full reproducibility?

No

Is the study design appropriate and is the work technically sound?

Partly

Are the conclusions drawn adequately supported by the results?

No

Are sufficient details of methods and analysis provided to allow replication by others?

No

Reviewer Expertise:

mosquito population genomics, entomology

I confirm that I have read this submission and believe that I have an appropriate level of expertise to confirm that it is of an acceptable scientific standard.

# Reviewer report: Reviewer response for version 2

**DOI:** 10.5256/f1000research.197074.r470431

**Published:** 2026-04-08

**Authors:** Arun Arumugaperumal

**Affiliations:** 1 Rajalakshmi Engineering College, Chennai, Tamil Nadu, India

The article reports the mitogenome sequence of *Philonis inermis*, a stem-galling weevil. The authors could have separated the mitochondria and could have done a mitogenome sequencing. Instead, they have sequenced the genomic DNA at low coverage and assembled the mitochondrial DNA. Then they have compared it with related insects and arrived at a phylogenetic tree. The absence of mitochondrial sequences from other members of the genus is a limitation. Given that, this sequence reported here will be of value to anybody working with the insect. 

 The authors can deposit the R-script used for analyses in a repository. The paragraph in discussion section starting with "The mitochondrial genome of *P. inermis* exhibits nucleotide a composition.." needs to be carefully checked. I think it is not conveying the intended meaning.

Is the work clearly and accurately presented and does it cite the current literature?

Yes

If applicable, is the statistical analysis and its interpretation appropriate?

Yes

Are all the source data underlying the results available to ensure full reproducibility?

Yes

Is the study design appropriate and is the work technically sound?

Yes

Are the conclusions drawn adequately supported by the results?

Yes

Are sufficient details of methods and analysis provided to allow replication by others?

Yes

Reviewer Expertise:

Genomics; Bioinformatics

I confirm that I have read this submission and believe that I have an appropriate level of expertise to confirm that it is of an acceptable scientific standard.

# Reviewer report: Reviewer response for version 2

**DOI:** 10.5256/f1000research.197074.r468385

**Published:** 2026-03-19

**Authors:** Michael J Raupach

**Affiliations:** 1 SNSB-Zoologische Staatssammlung München, Münchhausenstr, Munich, Germany

It is great to see that the authors have addressed all the suggestions and answered all the questions. From my perspective, therefore, I have no further objections to the publication of the manuscript.

Is the work clearly and accurately presented and does it cite the current literature?

Yes

If applicable, is the statistical analysis and its interpretation appropriate?

Yes

Are all the source data underlying the results available to ensure full reproducibility?

Yes

Is the study design appropriate and is the work technically sound?

Yes

Are the conclusions drawn adequately supported by the results?

Yes

Are sufficient details of methods and analysis provided to allow replication by others?

Yes

Reviewer Expertise:

DNA barcoding, molecular phylogenetics, mitochondrial genomics

I confirm that I have read this submission and believe that I have an appropriate level of expertise to confirm that it is of an acceptable scientific standard.

# Reviewer report: Reviewer response for version 1

**DOI:** 10.5256/f1000research.188059.r429006

**Published:** 2025-11-22

**Authors:** Michael J Raupach

**Affiliations:** 1 SNSB-Zoologische Staatssammlung München, Münchhausenstr, Munich, Germany

The manuscript “Characterization of the complete mitochondrial genome and evaluation of COI barcoding in Philonis inermis (Coleoptera: Curculionidae: Cryptorhynchinae) using genome skimming” by Clavijo-Giraldo and co-authors presents the complete mitochondrial genome of Philonis inermis, a Neotropical stem-galling weevil that is specialized on the invasive vine Passiflora foetida. Beside the characterization of the mitochondrial genome, the authors provide new DNA barcode data of 20 specimens from Colombia, analyzing the intra- and interspecific genetic divergence of this marker by combining the new data with already published sequences from GenBank/NCBI. In my eyes, the topic of this manuscript is interesting and for suitable for a publication in “F1000Reserach”. However, there are some points that should be added or discussed in a broader context (see below). 

 Without doubt, mitogenomes represent powerful phylogenetic markers. Drawbacks of using mitochondrial genomes in phylogenetic studies, however, include high substitution rates, leading to substitution saturation especially in deep evolutionary branches. In addition to the extracted BUSCO genes, it is quite easily to extract other useful multicopy nuclear genes as the rRNA and/or histone clusters from the given raw data as well. Whereas these genes are no focus-genes of the given study, they can become useful in further ongoing studies. Therefore, the authors should think about providing these popular phylogenetic marker genes as additional supplement, too.

 No contamination check has been done so far. I think that the amount of sequences of bacteria, fungi etc. will be very low, but nonetheless it should checked (e.g., using Kraken).

 The only use of mitogenomes in phylogenetic studies can have some serious limitations that should be mentioned/discussed (see above).

 In terms of the DNA barcode analysis, I recommend the creation of a project on the Barcode of Life Data System (BOLD; https://boldsystems.org/), the most popular workbench/sequence library for DNA barcode analysis, to (re)analyze the CO1 data set using the software tools offered there. This is especially true for the BIN approach (BOLD; Ratnasingham and Hebert (2013): PLOS ONE 8: e66213). Please check for already published sequences of closely related species on BOLD that should be included in such analysis. I feel that the assignment of a BIN will be very useful.

 Is this beetle able to fly? This can have a strong effect on its dispersal and therefore genetic structure as well.

 Other minor suggestions:

 It would be nice to present a photo of the weevil species if available (e.g., check iNaturalist).

 Where has the analyzed DNA been stored?

 Change "Cytochrome c oxidase subunit I" to "Cytochrome c (in italics) oxidase subunit I"

 A high AT-ratio is not only found in weevils but insects and arthropods in general. 

 What is a "shadow genome"?

Is the work clearly and accurately presented and does it cite the current literature?

Yes

If applicable, is the statistical analysis and its interpretation appropriate?

Yes

Are all the source data underlying the results available to ensure full reproducibility?

Yes

Is the study design appropriate and is the work technically sound?

Yes

Are the conclusions drawn adequately supported by the results?

Yes

Are sufficient details of methods and analysis provided to allow replication by others?

Yes

Reviewer Expertise:

DNA barcoding, molecular phylogenetics, mitochondrial genomics

I confirm that I have read this submission and believe that I have an appropriate level of expertise to confirm that it is of an acceptable scientific standard, however I have significant reservations, as outlined above.

# Response

**Published:** 2026-02-23

**Authors:** Andrés Gómez-Palacio

**Affiliations:** Universidad Pedagogica y Tecnologica de Colombia, Tunja, Boyaca, Colombia

Dr. Michael J Raupach

*We thank the reviewer for their positive evaluation and for recognizing the relevance of our study. We have carefully addressed all points raised and revised the manuscript accordingly. Detailed responses are provided below.*

 Without doubt, mitogenomes represent powerful phylogenetic markers. Drawbacks of using mitochondrial genomes in phylogenetic studies, however, include high substitution rates, leading to substitution saturation especially in deep evolutionary branches.

***Response:***

*We thank the reviewer for highlighting an important limitation of mitochondrial genomes in phylogenetic inference. We agree that despite their utility, high substitution rates can cause substitution saturation, particularly at deeper evolutionary timescales.*

*To address this, we have expanded the Introduction to explicitly acknowledge these constraints and to clarify that our study focuses on intraspecific and shallow-level phylogenetic resolution, where mitochondrial genomes and COI remain informative and widely used.*

*We have added the following text to the manuscript:*

*“Although mitogenomes are powerful markers, their use in phylogenetic studies is not without limitations. High substitution rates—particularly in third codon positions—can lead to substitution saturation in deep evolutionary branches, reducing the ability to accurately recover relationships among distantly related taxa. Such constraints are less problematic at shallow timescales, where mitochondrial genomes retain strong resolving power for population-level analyses and recent divergences. Because the present study focuses on intraspecific and closely related lineages, mitogenomic data remain well suited to our research objectives”*

 In addition to the extracted BUSCO genes, it is quite easily to extract other useful multicopy nuclear genes as the rRNA and/or histone clusters from the given raw data as well. Whereas these genes are no focus-genes of the given study, they can become useful in further ongoing studies. Therefore, the authors should think about providing these popular phylogenetic marker genes as additional supplement, too.

***Response*** *:*

*We thank the reviewer for this insightful suggestion. In addition to the 196 single-copy BUSCO orthologs already reported, we re-examined the BUSCO output (endopterygota_odb12) and identified 28 duplicated BUSCOs representing multicopy nuclear loci (now provided in Table S2). Furthermore, we extracted additional multicopy marker genes, including rRNA and histone clusters, directly from the assemblies (Table S3). These sequences have been deposited alongside the single-copy BUSCO set and are now explicitly referenced in the manuscript.*

*Although the present study focuses on the mitogenome and COI, we fully agree that these multicopy nuclear markers constitute a valuable resource for future phylogenomic or comparative genomic analyses. Accordingly, we have added a brief summary of these data in the Results section and clarified their potential utility in the Discussion.*

 No contamination check has been done so far. I think that the amount of sequences of bacteria, fungi etc. will be very low, but nonetheless it should checked (e.g., using Kraken).

***Response*** *:*

*We thank the reviewer for this suggestion. We have now performed a dedicated contamination assessment using Kraken2 (v2.1.2) with the PlusPF reference database. To specifically enrich for non-target sequences, quality-filtered reads were first mapped to the de novo assemblies, and unmapped read pairs were subsequently classified taxonomically. The analysis confirmed that bacterial, fungal, and viral sequences represent only a minor fraction of the reads in both libraries. Details of this analysis have been added to the Methods section, and the results are summarized in the revised manuscript.*

 The only use of mitogenomes in phylogenetic studies can have some serious limitations that should be mentioned/discussed (see above).

***Response*** *:*

*We appreciate the reviewer’s continued emphasis on this issue and fully agree that mitochondrial genomes alone have important limitations for phylogenetic inference, particularly due to substitution saturation at deeper evolutionary levels. We have revised the manuscript to explicitly state that mitogenomic data should not be interpreted as providing robust resolution of deep phylogenetic relationships. Instead, we clarify that their use in this study is restricted to intraspecific comparisons and shallow divergences, for which mitochondrial markers remain informative and widely applied. We also note that resolving deeper evolutionary relationships will require complementary nuclear genomic data, which is beyond the scope of the present study.*

 In terms of the DNA barcode analysis, I recommend the creation of a project on the Barcode of Life Data System (BOLD; https://boldsystems.org/), the most popular workbench/sequence library for DNA barcode analysis, to (re)analyze the CO1 data set using the software tools offered there. This is especially true for the BIN approach (BOLD; Ratnasingham and Hebert (2013): PLOS ONE 8: e66213). Please check for already published sequences of closely related species on BOLD that should be included in such analysis. I feel that the assignment of a BIN will be very useful.

***Response*** *:*

*We thank the reviewer for this valuable suggestion. Following this recommendation, we created a dedicated project in the Barcode of Life Data System (BOLD; project code PINE) and re-analyzed the complete COI dataset using the analytical tools available in the BOLD workbench. All COI sequences were uploaded and linked to vouchered specimens, including associated specimen images, and sequence validation was performed within BOLD.*

*Kimura 2-parameter (K2P) distance analyses and neighbor-joining clustering were conducted using BOLD’s distance summary and tree-building tools. These analyses confirmed extremely low intraspecific divergence among Philonis inermis sequences, consistent with our previous results based on pairwise K2P distances (0–0.006), and showed that all sequences form a single cohesive cluster.*

*We additionally surveyed BOLD for publicly available COI sequences of Philonis and closely related taxa within Cryptorhynchinae. No public barcode records for Philonis inermis or closely related congeners were found, indicating that the dataset generated here represents the first BOLD reference for this species. Consequently, no Barcode Index Number (BIN) was assigned by BOLD, which is expected for single-species datasets lacking comparable reference sequences and does not reflect a lack of genetic coherence. This behavior is consistent with the algorithmic nature of BIN assignment as described by Ratnasingham and Hebert (2013).*

*All barcode data and associated voucher information are available in BOLD under project code PINE, providing a baseline reference for future comparative and taxonomic studies of this genus.*

 Is this beetle able to fly? This can have a strong effect on its dispersal and therefore genetic structure as well.

***Response*** *:*

*We thank the reviewer for this observation. Philonis inermis is a poor flyer, and therefore active dispersal is expected to be limited. However, this species is a parasite associated with P. foetida, and its dispersal may occur passively via host movement, potentially facilitating connectivity among geographically separated populations. Consistent with this hypothesis, COI sequences from individuals collected across multiple Colombian localities (e.g., Antioquia and Córdoba) exhibited extremely low intraspecific divergence (minimal K2P distances), suggesting a genetically homogeneous population at the spatial scale examined. Nevertheless, the role of host-mediated dispersal remains hypothetical and should be further investigated using broader geographic sampling and additional nuclear markers. A brief sentence discussing this hypothesis and emphasizing that it requires further investigation has now been included in the Discussion section.*

 Other minor suggestions:

 It would be nice to present a photo of the weevil species if available (e.g., check iNaturalist).

***Response*** *:*

*We note that voucher-linked photographs of Philonis inermis are already available in the associated BOLD Systems project and correspond directly to some of the specimens analyzed in this study. Therefore, additional images from external sources (e.g., iNaturalist) were not included.*

 Where has the analyzed DNA been stored?

***Response*** *:*

 The total genomic DNA analyzed in this study is deposited in the entomological biobank of the Grupo de Investigación en Sistemática Molecular (GSM), Universidad Nacional de Colombia, Medellín, Colombia.

 Change "Cytochrome c oxidase subunit I" to "Cytochrome c (in italics) oxidase subunit I"

***Response*** *:*

*The term “Cytochrome c oxidase subunit I” has been corrected to “Cytochrome c oxidase subunit I” throughout the manuscript.*

 A high AT-ratio is not only found in weevils but insects and arthropods in general.

***Response*** *:*

*We have revised the text to clarify that the high A+T bias observed is a common feature of mitochondrial genomes not only in weevils but also across insects and arthropods in general.*

 What is a "shadow genome"?

***Response*** *:*

*The term “shadow genome” has been corrected to low-coverage genome sequencing (genome skimming) throughout the manuscript.*

# Reviewer report: Reviewer response for version 1

**DOI:** 10.5256/f1000research.188059.r429003

**Published:** 2025-11-22

**Authors:** Renee Ali

**Affiliations:** 1 johns Hopkins Bloomberg School of Public Health, Baltimore, Maryland, USA

Even though this manuscript offers useful genetic information about *Philonis inermis*, a number of sections need to be significantly revised before it can be accepted. The manuscript as it is currently worded suggests that the BUSCO was utilized to support the mitogenome's completeness; nevertheless, it should be made explicit that it was used to access the nuclear portion of the shallow sequence assemblies. Additionally, even though the study demonstrated that nuclear single copy orthologs can be accessed from low coverage sequences, this was given more attention than the mitochondrial genome, which is meant to be the primary emphasis as stated in the title. Additionally, the assemblies appear to be rather fragmented; the reasons why greater coverage was not attempted should be addressed. *Additionally, there are inconsistencies between the methods and results; for example no mention was made how phylogenetic analysis was done for complete mitogenomes only for the COI genes yet there is a tree represented in Figure 2 e which also lacks accession numbers*

 "This study presents the first complete mitochondrial genome for the genus *Philonis* and confirms the reliability of COI barcoding for its accurate identification. These genomic resources lay the foundation for integrative taxonomic, comparative, and evolutionary studies, and support the evaluation of *P. inermis* as a potential biological control agent against *P. foetida." - *I suggest editing statement , since there was no comparison or analysis of the other protein coding genes in the  mitochondrial genomes present to compare you cannot confirm reliability of COI for accurate ID, instead is useful for current status due to lack of other available information.

 Minor suggestions

 1.Use "shallow genome skimming" in place of "shadow-genome."

 2. Substitute "absence of a detectable trnI gene" for "deficiency of tRNA-Ile."

 3. When clarification is required, substitute "cryptorhynchine weevils" for "cryptorhynchine."

Is the work clearly and accurately presented and does it cite the current literature?

Partly

If applicable, is the statistical analysis and its interpretation appropriate?

Partly

Are all the source data underlying the results available to ensure full reproducibility?

No

Is the study design appropriate and is the work technically sound?

Partly

Are the conclusions drawn adequately supported by the results?

No

Are sufficient details of methods and analysis provided to allow replication by others?

No

Reviewer Expertise:

mosquito population genomics, entomology

I confirm that I have read this submission and believe that I have an appropriate level of expertise to confirm that it is of an acceptable scientific standard, however I have significant reservations, as outlined above.

# Response

**Published:** 2026-02-23

**Authors:** Andrés Gómez-Palacio

**Affiliations:** Universidad Pedagogica y Tecnologica de Colombia, Tunja, Boyaca, Colombia

Dr. Renee Ali

 We appreciate this observation and agree that the mitochondrial genome constitutes the primary focus of the study, as reflected in the title. In the revised manuscript, we have restructured the Results and Discussion sections to place clearer emphasis on mitogenome characterization, organization, nucleotide composition, and phylogenetic placement.

 The analysis of nuclear single-copy orthologs is now explicitly framed as a complementary outcome of the shallow genome-skimming approach rather than a central objective. While these nuclear markers provide valuable additional genomic resources, they are presented as secondary to the mitogenome assembly and COI barcoding analyses, which remain the core contributions of this work. ****We believe these revisions better align the manuscript structure with its stated primary emphasis.

 Additionally, the assemblies appear to be rather fragmented; the reasons why greater coverage was not attempted should be addressed.

***Response*:**

*We thank the reviewer for this important observation. The assemblies presented in this study were generated using a shallow genome-skimming strategy intentionally designed to recover the complete mitochondrial genome and representative nuclear markers (e.g., BUSCO orthologs), rather than to produce a high-contiguity nuclear genome assembly. Our primary objective was to establish foundational genomic resources for taxonomic validation and phylogenetic inference, for which moderate sequencing depth is sufficient.*

*As expected under this design, BUSCO analysis indicates partial recovery of the nuclear gene space, reflecting the limited coverage typical of genome-skimming approaches. Nevertheless, we successfully recovered 196 shared single-copy orthologs (191 with intact ORFs), as well as duplicated BUSCOs and multicopy rRNA and histone clusters, demonstrating that the data are sufficient for marker-based comparative and phylogenomic applications.*

*We agree that increased sequencing depth and incorporation of long-read technologies would substantially improve assembly contiguity and completeness. Such efforts are planned for future work but were beyond the scope of the present study. We have now clarified this rationale in the Discussion section to avoid ambiguity regarding the study design and objectives.*

 Additionally, there are inconsistencies between the methods and results; for example, no mention was made how phylogenetic analysis was done for complete mitogenomes only for the COI genes yet there is a tree represented in Figure 2 e which also lacks accession numbers

***Response*** *:*

 We thank the reviewer for identifying this inconsistency. The original submission did not explicitly describe the phylogenetic analysis performed using complete mitochondrial genome sequences, although it was presented in Figure 2e. We have now added a dedicated subsection in the Methods detailing the alignment procedure (MAFFT v7.525), model selection and maximum likelihood inference in IQ-TREE v2.0.3, and the use of 1,000 ultrafast bootstrap replicates. Additionally, GenBank accession numbers for all mitogenome sequences included in the analysis have been incorporated into the Methods section. These revisions resolve the methodological inconsistency and improve reproducibility.

 "This study presents the first complete mitochondrial genome for the genus *Philonis* and confirms the reliability of COI barcoding for its accurate identification. These genomic resources lay the foundation for integrative taxonomic, comparative, and evolutionary studies, and support the evaluation of *P. inermis* as a potential biological control agent against *P. foetida."* - I suggest editing statement, since there was no comparison or analysis of the other protein coding genes in the  mitochondrial genomes present to compare you cannot confirm reliability of COI for accurate ID, instead is useful for current status due to lack of other available information.

***Response*** *:*

*We thank the reviewer for this clarification. We agree that, given the absence of comparative analyses of other mitochondrial protein-coding genes, our data do not allow us to confirm the overall reliability of COI barcoding in a broader comparative framework. Accordingly, we have revised the statement to moderate the claim and to indicate that COI barcoding is useful for the current molecular identification of Philonis inermis under the limited availability of comparative genomic data.*

 Minor suggestions

1. Use "shallow genome skimming" in place of "shadow-genome."

***Response*** *:*

*Following the reviewer’s suggestion and previous comments from Dr. Michael J. Raupach, the term “shadow genome” has been replaced throughout the manuscript with “low-coverage genome sequencing” which more accurately reflects the sequencing approach used.*

  

1. Substitute "absence of a detectable trnI gene" for "deficiency of tRNA-Ile."

***Response*** *:*

*The phrase “deficiency of tRNA-Ile” has been revised to “absence of a detectable trnI gene” throughout the manuscript.*

  

1. When clarification is required, substitute "cryptorhynchine weevils" for "cryptorhynchine."

***Response*** *:*

*We thank the reviewer for this clarification. Throughout the manuscript, instances of the term “cryptorhynchine” have been revised to “cryptorhynchine weevils” where additional clarity was required,*
